# Evaluation of the antibacterial activity of a conventional orthodontic composite containing silver/hydroxyapatite nanoparticles

**DOI:** 10.1186/s40510-016-0153-x

**Published:** 2016-12-12

**Authors:** Ahmad Sodagar, Azam Akhavan, Ehsan Hashemi, Sepideh Arab, Maryam Pourhajibagher, Kosar Sodagar, Mohammad Javad Kharrazifard, Abbas Bahador

**Affiliations:** 1 Department of Orthodontics, Faculty of Dentistry, Tehran University of Medical Sciences, Tehran, Iran; 2 Tehran University of Medical Sciences, Tehran, Iran; 3 Radiation Applications Research School, Nuclear Science and Technology Research Institute, Tehran, Iran; 4 Department of Microbiology, Faculty of Medicine, Tehran University of Medical Sciences, Tehran, Iran; 5 Dental Research Center, Dentistry Research Institute, Tehran University of Medical Sciences, Tehran, Iran; 6 Laser Research Center, Dentistry Research Institute, Tehran University of Medical Sciences, Tehran, Iran; 7 Department of Microbiology, School of Medicine, Tehran University of Medical Sciences, Keshavarz Blvd., 100 Poursina Ave., Tehran, 14167-53955 Iran

**Keywords:** Antimicrobial activity, Hydroxyapatite, Nanoparticles, Orthodontic, Silver

## Abstract

**Background:**

One of the most important complications of fixed orthodontic treatment is the formation of white spots which are initial carious lesions. Addition of antimicrobial agents into orthodontic adhesives might be a wise solution for prevention of white spot formation. The aim of this study was to evaluate the antibacterial properties of a conventional orthodontic adhesive containing three different concentrations of silver/hydroxyapatite nanoparticles.

**Methods:**

One hundred and sixty-two Transbond XT composite discs containing 0, 1, 5, and 10 % silver/hydroxyapatite nanoparticles were prepared and sterilized. Antibacterial properties of these composite groups against *Streptococcus mutans*, *Lactobacillus acidophilus*, and *Streptococcus sanguinis* were investigated using three different antimicrobial tests. Disk agar diffusion test was performed to assess the diffusion of antibacterial agent on brain heart infusion agar plate by measuring bacterial growth inhibition zones. Biofilm inhibition test showed the antibacterial capacity of composite discs against resistant bacterial biofilms. Antimicrobial activity of eluted components from composite discs was investigated by comparing the viable counts of bacteria after 3, 15, and 30 days.

**Results:**

Composite discs containing 5 and 10 % silver/hydroxyapatite nanoparticles were capable of producing growth inhibition zones for all bacterial types. Results of biofilm inhibition test showed that all of the study groups reduced viable bacterial count in comparison to the control group. Antimicrobial activity of eluted components from composite discs was immensely diverse based on the bacterial type and the concentration of nanoparticles.

**Conclusions:**

Transbond XT composite discs containing 5 and 10 % silver/hydroxyapatite nanoparticles produce bacterial growth inhibition zones and show antibacterial properties against biofilms.

## Background

One of the most important complications of fixed orthodontic treatment is enamel demineralization [1]. Brackets and orthodontic accessories facilitate plaque accumulation and compromise oral hygiene maintenance which lead to an increase in oral bacteria count during orthodontic treatment [2–5]. Despite myriad progresses in orthodontics, fixed orthodontic treatment is still accompanied with a high risk of formation of white spot lesions which are found in more than 50% of orthodontic patients. Since white spots are unattractive and sometimes irreversible, they are a main concern for both orthodontists and orthodontic patients [1, 6–9].

Oral hygiene procedures in the frontline of caries prevention methods are not reliable enough due to dependence to patient cooperation. Therefore, one possible solution could be incorporation of antimicrobial agents into the orthodontic adhesives. In this regard, the addition of chlorhexidine and fluoride into adhesives has been suggested in some studies. However, the improper mechanical properties of the resultant adhesives as well as the short-term antimicrobial effects of these agents have made this approach questionable [10–14].

Application of nanotechnology in material science is a great step toward producing materials with enhanced chemical, mechanical, optical, and electrical features [15, 16]. Therefore, several studies have made efforts to evaluate antimicrobial and mechanical properties of various nanoparticles incorporated in orthodontic adhesives [14, 17–19].

Silver has long been used in medicine for its antimicrobial activity against various microorganisms. Recent studies have shown antibacterial properties of silver nanoparticles (AgNps) incorporated in poly(methyl methacrylate), dental composites, bonding agents, and resin-modified glass ionomers [17, 20–23]. Hydroxyapatite (HA) has been reported as an excellent carrier in AgNp production process [18]. In addition, HA nanoparticles have achieved brilliant successes in remineralization of incipient enamel lesions [24].

Considering unique features of AgNps and HA nanoparticles, the present study aimed at investigation of antibacterial effects of silver/hydroxyapatite (Ag/HA) nanoparticles incorporated in a conventional orthodontic adhesive composite (Transbond XT) against *Streptococcus mutans*, *Lactobacillus acidophilus*, and *Streptococcus sanguinis*.

## Methods

### Preparation of Ag/HA nanoparticles

The method of Akhavan et al. was used to synthesize Ag/HA nanoparticles [25]. Briefly, 100 mg of silver nitrate was dissolved in 20-ml deionized water. Afterwards, a solution of 1-g HA nanopowder (purchased from Aldrich Co.) in 80-ml distilled water was added to the sliver nitrate solution. The final mixture was stirred with magnetic mixer for 6 h, purged by nitrogen gas, and irradiated to 20-kGy dose of gamma ray in a Gamma Cell 220 (Nordion, Canada). The precipitation was centrifuged, washed, and dried overnight.

**Fig. 1:**
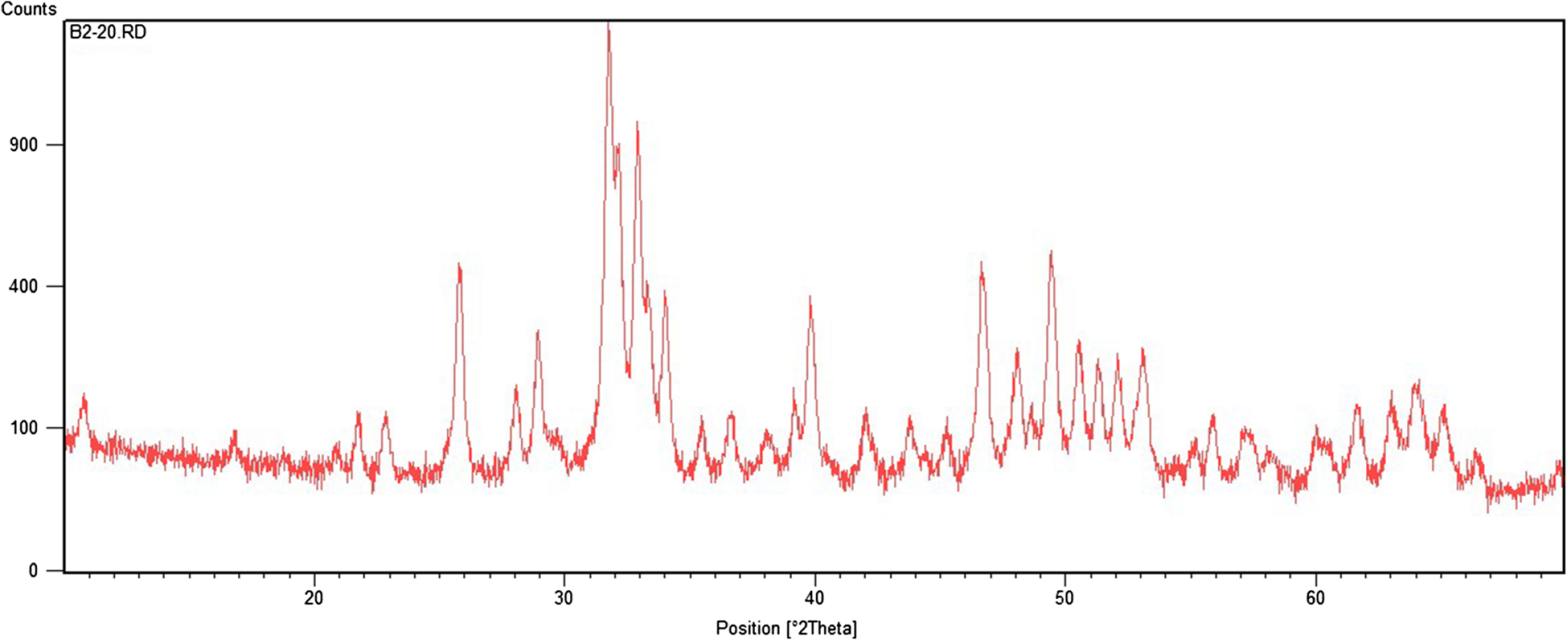
The XRD spectrum of irradiated sample at 20 kGy presents peaks at 2*θ* of 37.81, 44.21, and 63.41, assigned to the diffractions from the (1 1 1), (2 0 0), and (2 2 0) planes of face-centered cubic (fcc) silver nanoparticles

The formation of silver nanoparticles incorporated in the HA composite was studied by the XRD pattern of the Ag/HA sample using a Holland Philips Xpert X-ray diffractometer (CuKα) (Fig. 1).

**Fig. 2:**
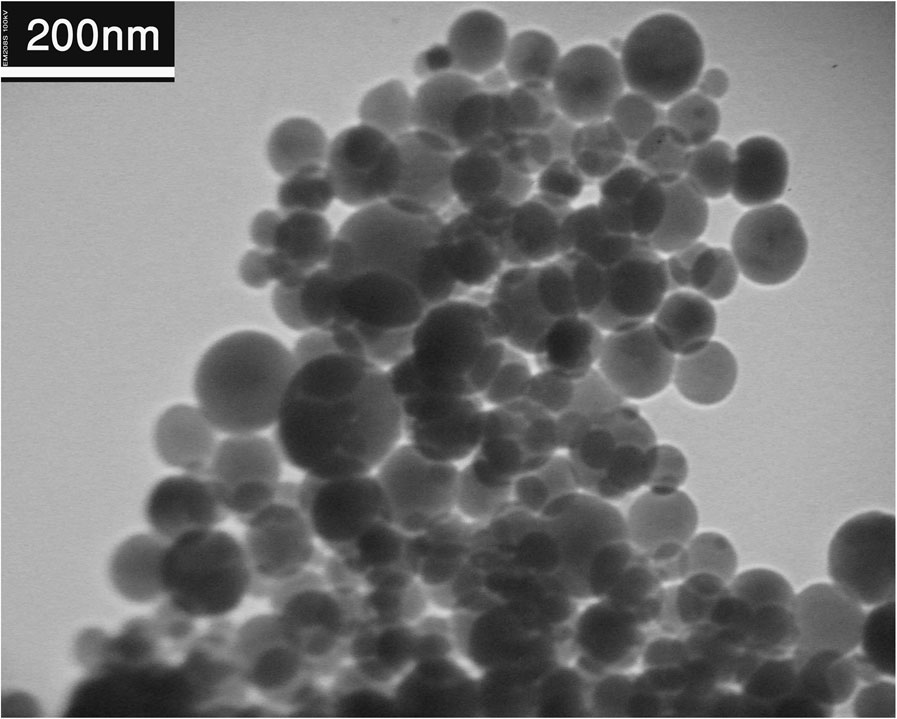
TEM image shows formation of nanosize particles

The TEM morphology of Ag/HA sample was evaluated on a Phillips EM 208S electron microscope operating at acceleration voltage of 100 kV (Fig. 2). As observed, the Ag/HA particles were nanosize and non-aggregated form, showing that the producing process had no effect on the initial HA particle distribution. From the figure, the majority of particles in Ag/HA powder were of spherical shape, with a mean diameter range of 55–65 nm.

### Preparation of composite discs

A total number of 162 composite discs were used in this study. After preparation of Ag/HA nanoparticles, Transbond XT pastes (3 M Unitek, CA, USA) with 1, 5, and 10 % (*w*/*w*) Ag/HA nanoparticles were produced by mixing proper amounts of nanoparticles into the composite paste precisely. Pure Transbond XT composite discs were used as control group. In order to obtain specimens with dimensions close to bracket bases, 5-mm diameter circular metal molds were filled with composites and covered by glass slides. After light curing for 20 s from each side (Bluphase® 16i, Ivoclar Vivadent, AG, Australia), composite discs were expelled from the molds and sterilized via gamma ray.

### Preparation of bacterial suspensions

Lyophilized *S. mutans* (ATCC 25175) and *S. sanguinis* (ATCC 10556) were rehydrated in brain-heart infusion (BHI) broth (Difco, Sparks, MD, USA) in 5 % CO_2_ atmosphere at 37 °C for 48 h. Microbial suspensions with 10^8^ CFU/ml were prepared using spectrophotometer. Optical density of 0.2 correspond to 10^8^ cells/ml. Lyophilized *L. acidophilus* (ATCC 4356) was grown in BHI broth in anaerobic conditions at 37 °C. For *L. acidophilus,* an optical density equal to 1 corresponds to 10^8^ cells/ml.

### Disk agar diffusion test (DAD)

DAD determines the ability of antibacterial agents to diffuse within agar and produce bacterial inhibition zone. Twenty microliters from bacterial suspensions was spread on the cation-adjusted Mueller Hinton agar (CAMHA; Himedia, India) plate via a sterilized swap, and composite discs were placed on the surface of plates with 2-cm distance from each other. Plates containing *L. acidophilus* were incubated anaerobically, while other plates were incubated in capnophilic condition. Following incubation for 48 h, the growth inhibition zones were measured.

### Biofilm inhibition test

Biofilms were formed on composite discs by inoculation of bacterial suspensions in composite discs in flat-bottom 96-well microtiter plates (TPP; Trasadingen, Switzerland) and incubation at 37 °C for 72 h. Afterwards, composite discs were rinsed thoroughly with sterilized saline to wash away the planktonic and loosely attached cells. Finally, in order to dislodge biofilms, composite discs were sonicated at 50 Hz in 150 W and vortexed for 1 min. The CFUs/ml of test wells was calculated using Miles and Misra method [26].

### Antibacterial properties of eluted components

In order to evaluate the antibacterial activity of the eluted components from composite discs, the specimens were placed in tubes containing 5-ml BHI broth at 37 °C in a dark environment. After 3, 15, and 30 days, discs were removed and liquid medias were transferred to new plastic tubes. Fifty microliters of bacterial suspension (in final concentration 2.5 × 10^5^ CFU/ml) was added to the latter tube, and tubes were agitated at 300 rpm for 24 h at 37 ° C. The CFUs/ml of test wells was calculated using Miles and Misra method [26].

### Statistical analysis

Kruskal–Wallis test, ANOVA, and Tukey HSD test were used for statistical analysis. *P* < 0.05 was considered statistically significant.

## Results

### Disk agar diffusion test (DAD)

Results of DAD test indicated that adding 1 % Ag/HA nanoparticles to composite does not produce bacterial inhibition zone for any of bacterial strains. However, composite discs containing 5 and 10 % nanoparticles showed bacterial inhibition halos which did not have significantly different diameters. The complete results of DAD test are presented in Table 1.

**Table 1:** Average diameters of bacterial growth inhibition zones (mm)

Bacterial Type	Concentration Ag/HA Nanoparticles (%)	Diameter (mm)
*S. mutans*	0	0
	1	0
	5	6.33 ± 0.58
	10	8.66 ± 1.15
*S. sanguinis*	0	0
	1	0
	5	7.66 ± 1.15
	10	9.66 ± 1.52
*L. acidophilus*	0	0
	1	0
	5	5.66 ± 0.58
	10	7.66 ± 0.58

### Biofilm inhibition test

**Fig. 3:**
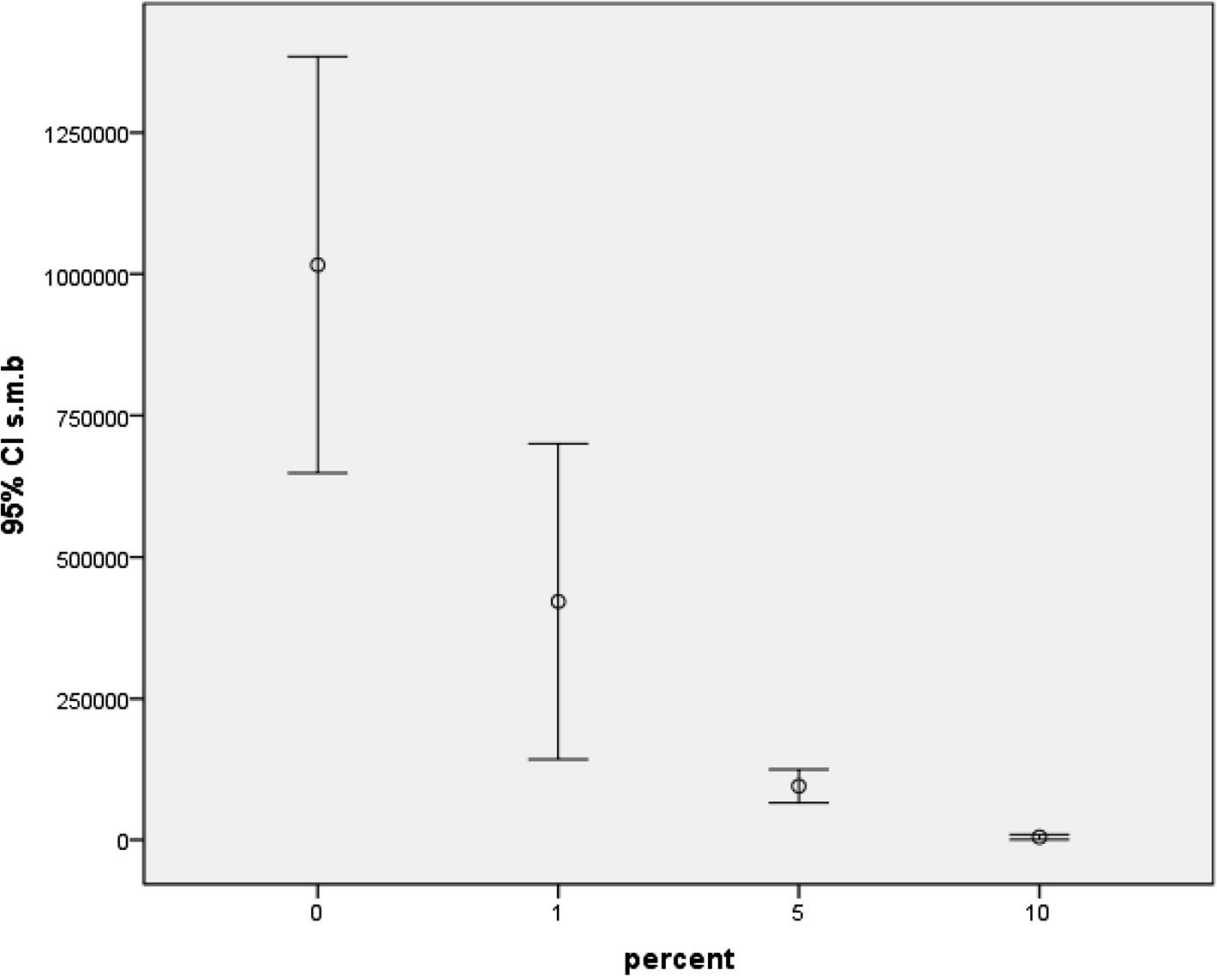
Viable counts of *S. mutans* biofilms on composite discs containing 0, 1, 5, and 10 % Ag/HA nanoparticles

**Fig. 4:**
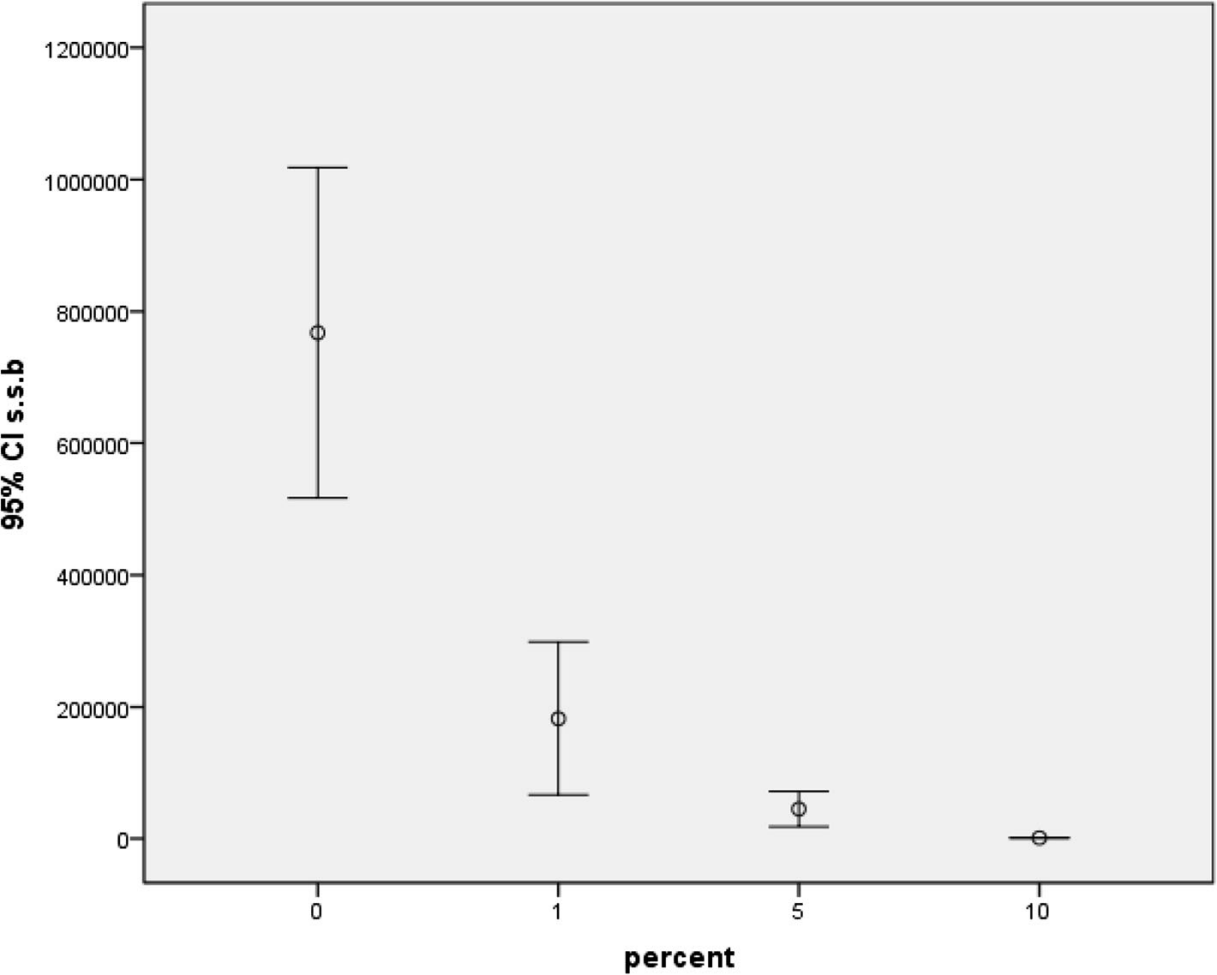
Viable counts of *S. sanguinis* biofilms on composite discs containing 0, 1, 5, and 10 % Ag/HA nanoparticles

**Fig. 5:**
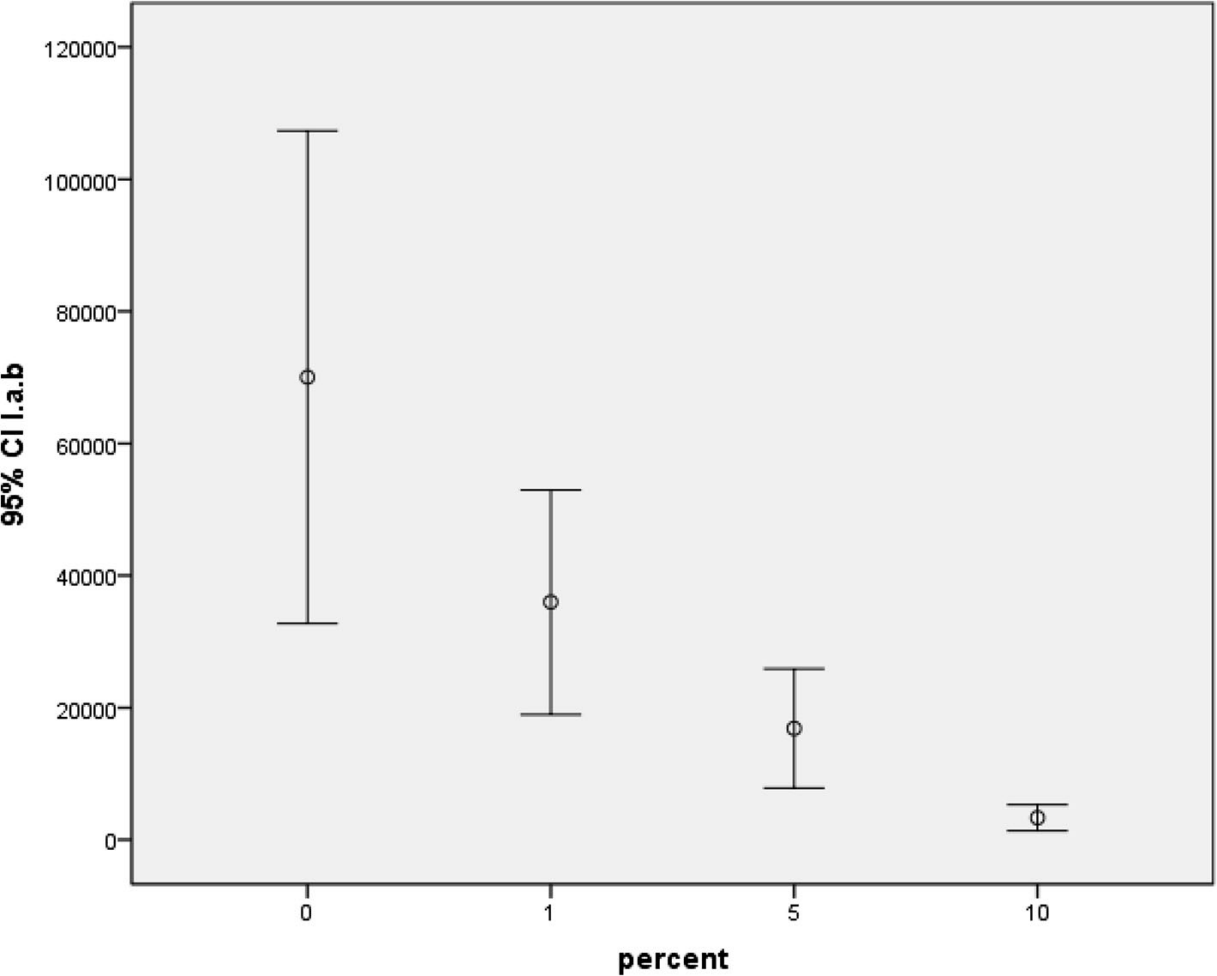
Viable counts of *L. acidophilus* biofilms on composite discs containing 0, 1, 5, and 10 % Ag/HA nanoparticles

Investigation of the antibacterial effect of the study groups against mature *S. mutans* biofilm revealed significant differences between all groups except between the 5 and 10 %. Results of biofilm inhibition test for *S. sanguinis* and *L. acidophilus* were similar and for both bacterial strains, significant differences between all groups except between 1 and 5 % as well as between 5 and 10 % were recorded. Error bars in Figs. 3, 4, and 5 depict the results of biofilm inhibition test for *S. mutans*, *S. sanguinis*, and *L. acidophilus*, respectively.

### Eluted component test

Results of the eluted component test for *S. mutans* in days 15 and 30 showed that with increase in concentration of nanoparticles, logarithm of colony count decreases with statically significant differences between all groups. Logarithms of colony count of *S. sanguinis* were not significantly different between groups 1 and 5 % in any day. However, composite discs with 10 % nanoparticles decreased the colony count of *S. sanguinis* significantly.

Reduction of colony count of *L. acidophilus* in day 3 was significantly different between all groups, while difference between groups 1 and 5 % in days 15 and 30 was not statistically significant.

Results of the eluted components test are presented in Table 2.

**Table 2:** Logarithm of bacterial count in liquid media after 3, 15, and 30 days

Bacterial type	Incubation time (day)	Percentage of Ag/HA nanoparticles			
		0 %	1 %	5 %	10 %
*Streptococcus mutans* log	0	8.371 ± 0.027			
	3		8.281 ± 0.202	8.184 ± 0.036	7.885 ± 0.073
	15		8.312 ± 0.010	8.241 ± 0.027	8.041 ± 0.033
	30		8.334 ± 0.029	8.268 ± 0.014	8.150 ± 0.020
*Streptococcus sanguinis* log	0	8.346 ± 0.049			
	3		8.233 ± 0.031	8.115 ± 0.014	7.777 ± 0.073
	15		8.254 ± 0.038	8.209 ± 0.020	8.096 ± 0.029
	30		8.300 ± 0.014	8.278 ± 0.020	8.187 ± 0.031
*Lactobacillus acidophilus* log	0	8.295 ± 0.035			
	3		8.248 ± 0.036	8.160 ± 0.032	7.996 ± 0.008
	15		8.271 ± 0.027	8.228 ± 0.009	8.112 ± 0.053
	30		8.285 ± 0.017	8.270 ± 0.011	8.228 ± 0.021

## Discussion

Current concerns about white spots formation during fixed orthodontic treatment have led to conduction of several studies aiming at induction of antibacterial properties in orthodontic adhesives by using various nanoparticles such as ZnO, TiO_2_, Ag, and polyethylenimine [14, 17, 19, 27].

Selection of HA nanoparticles in the current study was based on two major reasons. Firstly, the HA nanoparticles have shown the potential of remineralizing initial enamel lesions [28]. Secondly, we used the capability of HA in acting as a solid support in the nanoparticles’ production procedure [25].

Several methods have been suggested for synthesis of silver-doped hydroxyapatite nanoparticles. We applied gamma irradiation for production of silver nanoparticles and used HA as carrier. This method is capable of producing pure composite material in which silver nanoparticles are dispersed homogeneously [25].

Results of biofilm inhibition test revealed that all three concentrations of Ag/HA nanoparticles decrease the colony count of all bacteria species significantly, whereas increasing the concentration of Ag/HA nanoparticles from 5 to 10 % does not show any significant reductions. The biofilm inhibition test is immensely important because biofilms are much more resistant to antimicrobial agents in comparison to planktonic cells [29]. Although *S. mutans* is the main bacteria associated with initial carious lesions such as white spots [30–32], *S. sanguinis* is associated with non-cariogenic plaque and is in competition with *S. mutans* [33, 34]. Therefore, orthodontic adhesives containing 5 % Ag/HA nanoparticles are highly efficient against white spot formation because in comparison to adhesive with 1% Ag/HA nanoparticles, they show increased antibacterial activity against *S. mutans* without significantly higher effect against *S. sanguinis*.

The same result was obtained by eluted component test about the logarithms of colony counts of *S. sanguinis* and *S. mutans* on the 15th and 30th days. Therefore, similar to the biofilm inhibition test, eluted component test also supports adding 5 % Ag/HA nanoparticles to orthodontic adhesives.

Regarding results of DAD test, unlike 1 % Ag/HA nanoparticles, incorporation of 5 % Ag/HA nanoparticles produces growth inhibition zone for all three bacteria. Furthermore, increasing nanoparticles from 5 to 10 % does not change the inhibition zone significantly. Therefore, DAD test as well as the two previous tests indicates that adhesives containing 5 % Ag/HA nanoparticles provide proper antibacterial features.

Although adding Ag/HA nanoparticles to resin composite brings about brilliant antibacterial properties, the possible adverse effects on mechanical features should not be overlooked. Our previous study revealed that incorporation of 5 % Ag/HA nanoparticles to orthodontic bonding resin does not compromise shear bond strength, while adding 1 and 10 % Ag/HA nanoparticles increases and decreases it, respectively [18]. Therefore, results of the present study in combination with our previous research indicate that orthodontic adhesives containing 5 % Ag/HA nanoparticles provide suitable features from both antimicrobial and mechanical aspects.

## Conclusions

Combination of the results of three antimicrobial tests showed that adding 5 % Ag/HA nanoparticles to orthodontic adhesives reduces growth of cariogenic bacteria, has less effect against non-cariogenic *S. sanguinis*, and is capable of producing bacterial growth inhibition zone.

## Abbreviations

Ag/HA: Silver hydroxyapatite
AgNps: Silver nanoparticles
HA: Hydroxyapatite

## References

[1] Derks A, Katsaros C, Frencken JE, van't Hof MA, Kuijpers-Jagtman AM (2004). Caries-inhibiting effect of preventive measures during orthodontic treatment with fixed appliances. A systematic review. Caries Res.

[2] Freitas AO, Marquezan M, Nojima Mda C, Alviano DS, Maia LC (2014). The influence of orthodontic fixed appliances on the oral microbiota: a systematic review. Dental Press J Orthod.

[3] Maret D, Marchal-Sixou C, Vergnes JN (2014). Effect of fixed orthodontic appliances on salivary microbial parameters at 6 months: a controlled observational study. J Appl Oral Sci.

[4] Peros K, Mestrovic S, Anic-Milosevic S, Slaj M (2011). Salivary microbial and nonmicrobial parameters in children with fixed orthodontic appliances. Angle Orthod.

[5] Sukontapatipark W, El‐Agroudi MA, Selliseth NJ, Thunold K, Selvig KA (2001). Bacterial colonization associated with fixed orthodontic appliances. A scanning electron microscopy study. Eur J Orthodontics.

[6] Gorelick L, Geiger AM, Gwinnett AJ (1982). Incidence of white spot formation after bonding and banding. Am J Orthod.

[7] Sundararaj D, Venkatachalapathy S, Tandon A, Pereira A (2015). Critical evaluation of incidence and prevalence of white spot lesions during fixed orthodontic appliance treatment: a meta-analysis. J Int Soc Prev Community Dent.

[8] Lucchese A, Bertacci A, Chersoni S, Portelli M (2012). Primary enamel permeability: a SEM evaluation in vivo. Eur J Paediatr Dent.

[9] Lucchese A, Gherlone E (2013). Prevalence of white-spot lesions before and during orthodontic treatment with fixed appliances. Eur J Orthod.

[10] Cohen WJ, Wiltshire WA, Dawes C, Lavelle CL (2003). Long-term in vitro fluoride release and rerelease from orthodontic bonding materials containing fluoride. Am J Orthod Dentofacial Orthop.

[11] Jedrychowski JR, Caputo AA, Kerper S (1983). Antibacterial and mechanical properties of restorative materials combined with chlorhexidines. J Oral Rehabil.

[12] Paschos E, Kurochkina N, Huth KC, Hansson CS, Rudzki-Janson I (2009). Failure rate of brackets bonded with antimicrobial and fluoride-releasing, self-etching primer and the effect on prevention of enamel demineralization. Am J Orthod Dentofacial Orthop.

[13] Ribeiro J, Ericson D (1991). In vitro antibacterial effect of chlorhexidine added to glass-ionomer cements. Scand J Dent Res.

[14] Poosti M, Ramazanzadeh B, Zebarjad M, Javadzadeh P, Naderinasab M, Shakeri MT (2013). Shear bond strength and antibacterial effects of orthodontic composite containing TiO2 nanoparticles. Eur J Orthodontics.

[15] Mitra SB, Wu D, Holmes BN (2003). An application of nanotechnology in advanced dental materials. J Am Dent Assoc.

[16] Thomas J (2006). An introduction to nanotechnology: the next small big thing. Development.

[17] Ahn SJ, Lee SJ, Kook JK, Lim BS (2009). Experimental antimicrobial orthodontic adhesives using nanofillers and silver nanoparticles. Dent Mater.

[18] Akhavan A, Sodagar A, Mojtahedzadeh F, Sodagar K (2013). Investigating the effect of incorporating nanosilver/nanohydroxyapatite particles on the shear bond strength of orthodontic adhesives. Acta Odontol Scand.

[19] Aydin Sevinc B, Hanley L (2010). Antibacterial activity of dental composites containing zinc oxide nanoparticles. J Biomed Mater Res B Appl Biomater.

[20] Cheng L, Zhang K, Melo MA, Weir MD, Zhou X, Xu HH (2012). Anti-biofilm dentin primer with quaternary ammonium and silver nanoparticles. J Dent Res.

[21] Wang X, Wang B, Wang Y (2015). Antibacterial orthodontic cement to combat biofilm and white spot lesions. Am J Orthod Dentofacial Orthop.

[22] Sodagar A, Kassaee MZ, Akhavan A, Javadi N, Arab S, Kharazifard MJ (2012). Effect of silver nano particles on flexural strength of acrylic resins. J Prosthodont Res.

[23] Morones JR, Elechiguerra JL, Camacho A (2005). The bactericidal effect of silver nanoparticles. Nanotechnology.

[24] Hannig C, Hannig M (2010). Natural enamel wear—a physiological source of hydroxyapatite nanoparticles for biofilm management and tooth repair?. Med Hypotheses.

[25] Akhavan A, Sheikh N, Khoylou F, Naimian F, Ataeivarjovi E (2014). Synthesis of antimicrobial silver/hydroxyapatite nanocomposite by gamma irradiation. Radiat Phys Chem.

[26] Miles AA, Misra SS, Irwin JO (1938). The estimation of the bactericidal power of the blood. J Hyg (Lond).

[27] Beyth N, Houri-Haddad Y, Baraness-Hadar L, Yudovin-Farber I, Domb AJ, Weiss EI (2008). Surface antimicrobial activity and biocompatibility of incorporated polyethylenimine nanoparticles. Biomaterials.

[28] Huang SB, Gao SS, Yu HY (2009). Effect of nano-hydroxyapatite concentration on remineralization of initial enamel lesion in vitro. Biomed Mater.

[29] Choi O, Yu CP, Esteban Fernandez G, Hu Z (2010). Interactions of nanosilver with Escherichia coli cells in planktonic and biofilm cultures. Water Res.

[30] Gaines S, James T, Folan M, Baird A, O'Farrelly C (2003). A novel spectrofluorometric microassay for Streptococcus mutans adherence to hydroxylapatite. J Microbiol Methods.

[31] Hernández-Sierra JF, Ruiz F, Pena DCC (2008). The antimicrobial sensitivity of Streptococcus mutans to nanoparticles of silver, zinc oxide, and gold. Nanomedicine.

[32] Li J, Helmerhorst E, Leone C (2004). Identification of early microbial colonizers in human dental biofilm. J Appl Microbiol.

[33] Becker MR, Paster BJ, Leys EJ (2002). Molecular analysis of bacterial species associated with childhood caries. J Clin Microbiol.

[34] Kreth J, Merritt J, Shi W, Qi F (2005). Competition and coexistence between Streptococcus mutans and Streptococcus sanguinis in the dental biofilm. J Bacteriol Nov.

